# How Blockchain Will Change Leadership Strategies for Effectively Managing Organizational Change

**DOI:** 10.3389/fpsyg.2022.907586

**Published:** 2022-06-02

**Authors:** Richard Richard, Harjanto Prabowo, Agung Trisetyarso, Benfano Soewito

**Affiliations:** ^1^Information Systems Department, School of Information Systems, Bina Nusantara University, Jakarta, Indonesia; ^2^Bina Nusantara Graduate Program, Computer Science Department, Bina Nusantara University, Jakarta, Indonesia

**Keywords:** blockchain, leadership, change leader effectiveness, organizational change, strategy

## Introduction

Blockchain technology is becoming a gold standard in maintaining data integrity through decentralized data stores. A smart contract allows people to build an application on a decentralized server. This fact has an immediate impact on several industries. Anyone can instantly access global liquidity in the financial sector by using decentralized finance. Artists and game developers can mint a Non-Fungible Token (NFT) that represents digital collectibles or in-game items in the entertainment and game industry. These phenomena become a new horizon for creating a system that can reach global users instantly. Blockchain also impacts the fundraising process. People can access international funds by introducing their project and offering tokens to represent its ownership. In its early days, a fundraising mechanism through a blockchain is known as Initial Coin Offering (ICO) (Kondova and Simonella, 2019). Technology causes disruption, not only in the system structure, but also in talent. The Industrial Revolution 4.0 causes an increasing need for qualified digital talent by every organization (Fahlevi, 2020). Moreover, in the current pandemic era, technological adaptation is becoming increasingly massive. So, it is important for digital leaders to pay attention to this aspect so that the organization can go further (Fahlevi et al., 2019). Talking about talent, of course there are skills needed to respond to the challenges of the times. The latest system such as the Security Token Offering (STO) used by the company is one of the blockchain adoptions that is enough to change the foundation of organizational behavior in modern times to become decentralized.

In the old way the organization will raise funds in a traditional way so that the leadership will stick to the old ways such as releases on the stock exchange and using traditional crowdfunding. At this time, along with changing times, it has also changed organizational behavior, especially the behavior of leaders in terms of raising funds for companies. STO adoption bridges the gap between ICO and Initial Public Offering (IPO). Thus, STOs offer a safer way to raise funds publicly, but still comply with existing regulations and provide easy digital access. In ICO, the token issuer publishes a “white paper” (document like a business plan) that elaborates the project's detail (Momtaz et al., 2019). People can participate in the offering by sending cryptocurrency to the token issuer's address and getting the token as an exchange. ICO reached its popularity with raised more than $18B over the past 2017 and 2018 (Fenu et al., 2018). However, the easiness of fundraising through ICO led this innovation to many scam activities. ICO advisory firm Satis Group reports that approximately 78% of ICOs are scams, around 4% fail, and only 15% subsequently traded on an exchange (Dowlat and Hodapp, 2018). This fact leads several countries to ban ICO activities and warns citizens to consider ICO a very high-risk investment. However, the scam and ban of ICO are not relieving the fact that ICO is the most promising fundraising method for anyone that does not have access to VC and global investors. Several firms proposed a new mechanism called STO. A token offering process should sit under applicable securities and fundraising laws.

An STO is essentially a regulated version of an ICO. It involves the creation of digital assets called security tokens. STO could be especially run on a custom-made blockchain platform for the transaction or use an existing system, such as Ethereum or Hyperledger. The blockchain platform also includes transfer and other controls to enable regulatory compliance. The token issuers must comply with relevant legal and regulatory requirements, as do brokers and exchanges. The issues related to transferability, electronic transactions obligations, custody regulation, insurance, and stamp duty can also arise.

The benefits and application of blockchain technology in Indonesia have attracted the attention of a number of parties to adopt the technology. Ironically until now, especially for the real sector, blockchain optimization is faced with a number of obstacles. In addition to lack of understanding, synergism is a factor that influences this. The leadership factor is also important because without a change in management initiated by the leadership to change their strategy to keep up with the times, the adoption of blockchain technology will not be rapid.

The motivation of this paper arises from the fact that the STO mechanism could allow its stakeholders to reach the infinite potential of a token offering process without ignoring the regulatory barriers in each country. The existence of a decentralized record of a smart contract allows the token to be exchanged quickly. This research aims to design an STO system to fit the regulatory requirements while preserving decentralization. With Blockchain, people can benefit from global liquidity access and native data integration in the token offering process.

## Blockchain

**Figure 1:**
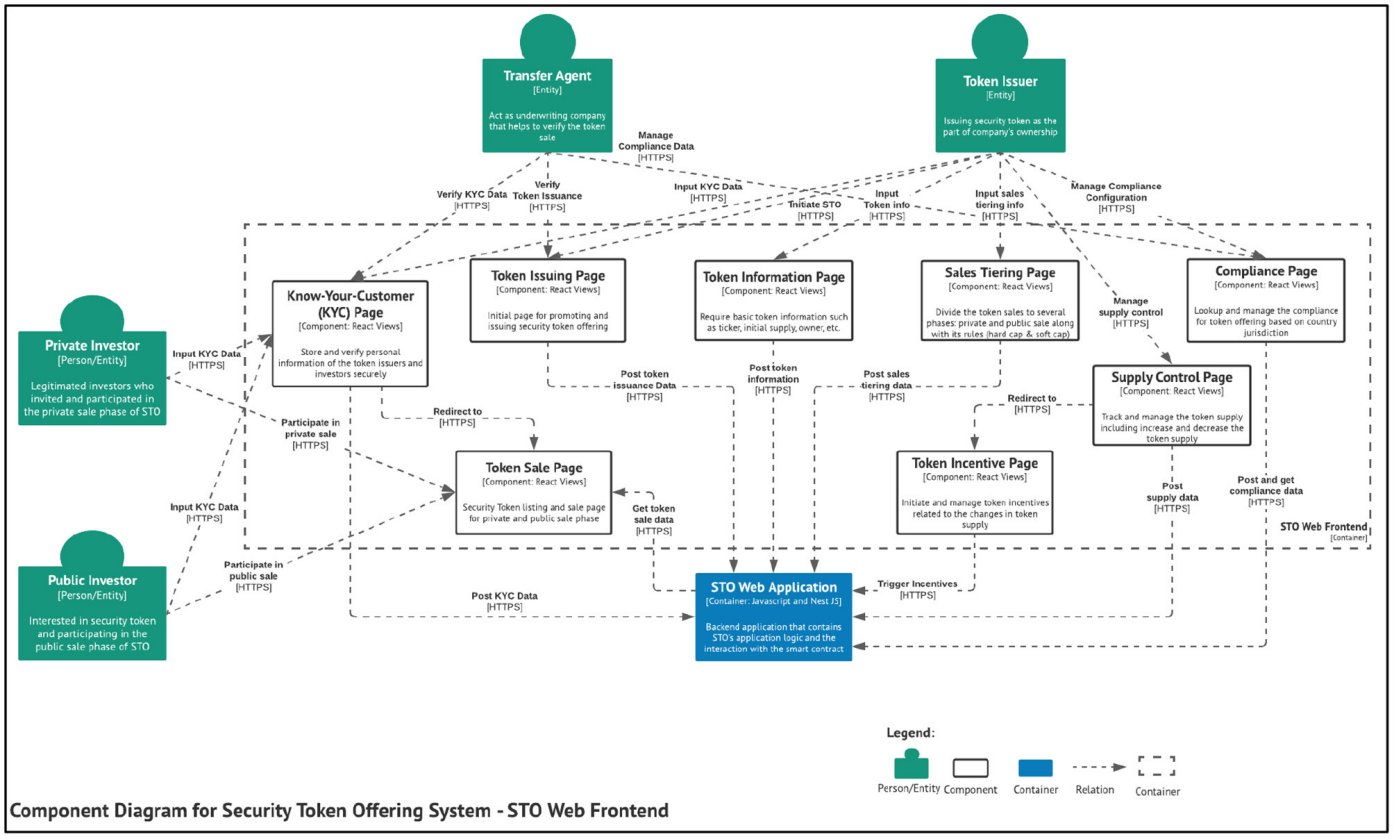
Component diagram for STO web frontend.

With the popular definition, Blockchain is a linked list of blocks (nodes) that contains a set of information. This linked list forms a network chain owned by each participant (miners). The mechanism that decides which miners will be authorized to validate a new block is called the blockchain consensus mechanism (e.g., proof-of-work, proof-of-stake). Blockchain technology is an immutable, transparent, and traceable platform that could answer the utmost trust, which a standard centralized architecture system does not have. Figure 1 shows the chain-structured Blockchain, which has become the “gold standard” many blockchain networks and projects use.

## Security Token Offering

The Security Token Offering (STO) was coined as an unregulated Initial Coin Offering (ICO) solution. The token in STO represents a company's ownership and sits under applicable securities regulation (Yano et al., 2020). STO mechanism usually has a more straightforward procedure than the capital market's current capital-raising process (Ante and Fiedler, 2019). The adoption of security tokens in the financial industry allows investors to fraction the digital asset ownership while easily tracking the ownership and transfer of an asset (Levin et al., 2018). By creating a security token, investors can access a broader market and generate more liquidity for the capital-raising process (Liu and Wang, 2019). A well-designed STO will significantly benefit the fundraising process (Narayan and Tidström, 2020).

In designing an STO, investors' rights and obligations must be included in the security token, whether as a code or procedure (Miglo, 2021). The exchanges-related regulations are also considered essential components that need to be included in the STO mechanism (Kondova and Simonella, 2019). Several more features and functions are considered crucial factors to be defined in the STO. First, the information of token issuers, potential investors, and jurisdictions needs to be disclosed during the STO process (Venegas, 2017; Martino et al., 2019). Second, the government's support, supervision, and verification also become a vital factor in ensuring the accountability of the STO process (Chohan, 2019; Momtaz et al., 2019). Finally, the standardization and synergy between the regulator, token issuer, and every stakeholder in STO also become an essential agenda in the future (Van der Elst and Lafarre, 2019; Zhang et al., 2019).

## System Architecture

In designing the system architecture, stakeholders and existing software systems involved in the STO are identified. The stakeholders involved in the STO mechanism are identified as follows.

- **Token issuer**: Issuing security tokens as part of the company's ownership.
- **Transfer agent**: Act as an underwriting company that helps to verify the token sale.
- **Public investor**: Party interested in security tokens and participating in the public sale phase of STO.
- **Private investor**: The party invited and participated in the private sale phase of STO.
- **Security auditor**: Third-party entity responsible for giving audits and checking security vulnerabilities.

Furthermore, the existing software system is identified as follows.

- **Regulation and compliance system**: System that provides applicable securities and fundraising laws.
- **Rating system**: Software system owned by a rating agency to give the rating to security token based on the token performance and information.
- **Custody system**: Storing and managing security tokens on the crypto wallet secured through several mechanisms such as HD Wallet, HSM, and MPC.
- **Third-party data provider system**: Data provider for smart contract (e.g., price data, historical data).
- **Decentralized file storage system**: Decentralized system for storing and accessing files.
- **Blockchain platform**: Decentralized public/permissioned ledger that can record and execute the transaction with a set of algorithms.

With the stakeholders and existing systems identified, this paper proposes the system architecture for STO. The first phase of modeling is the system context diagram.

## System Context Diagram

The diagram will show high-level interaction between proposed systems, stakeholders, and existing systems. This paper proposes two core software systems in the STO mechanism illustrated in the system context diagram. The two software systems consist of:

- **Security Token Offering System**: A Web2 or Common App including Backend and Frontend application.
- **Security Token Offering DApp System**: A Web3 or Decentralized App including Smart Contract and Decentralized Storage.

Both systems interact with stakeholders and existing systems in the STO mechanism. The proposed system context diagram becomes the big picture for the following diagram.

## Component Diagram

This section provides a more in-depth visualization of the STO system. The component represents a grouping of related functionality encapsulated behind a well-defined interface. The first component diagram proposed in this paper is the STO web frontend. The components are coined based on the essential token offering functions explained in the previous section.

The component diagram for the STO web frontend visualizes the interaction between components and its interaction protocols. The components in the STO web frontend include:

- **Know-Your-Customer (KYC) page**: Store and securely verify the token issuers' and investors' personal information.
- **Token issuing page**: Initial page for promoting and issuing security token offering.
- **Token information page**: Require basic token information such as ticker, initial supply, owner, etc.
- **Token sale page**: Security Token listing and sale page for private and public sale phase.
- **Sales tiering page**: Divide the token sales into several phases: private and public sales along with its rules (hard cap and soft cap).
- **Supply control page**: Track and manage the token supply, including increasing and decreasing the token supply.
- **Token incentive page**: Initiate and manage token incentives related to the changes in token supply.
- **Compliance page**: Lookup and manage the compliance for token offering based on country jurisdiction.

These components represent a static web frontend for handling several activities related to the STO mechanism. Most of the stakeholders will interact with the STO system through these components. The diagram also shows the interaction between components and the STO web application. This paper proposes a component diagram for the STO web application to extend the details of the container.

## Conclusion

The outcome of this paper is a novel system architecture of an STO system. The architecture and design were coined to fulfill the basic requirement of token offering and applicable regulations and laws while preserving decentralization. As mentioned in section System Architecture, the basic consideration in designing the system architecture is the token offering requirements found in the previous research. This paper uses the C4 model approach to design the system architecture to address the requirements. The system context diagram shows that the STO architecture is designed as STO System (off-chain) and STO DApp System (on-chain). Off-chain is a system that runs traditionally but is designed to interact with the blockchain system. An On-chain system is a system that runs and is hosted on a blockchain platform. Both systems are complementary to each other in running the STO mechanism. In the system component diagram, basic token offering requirements are represented by several STO web applications and STO web frontend components. The component diagram also explains the security token's security aspect by visualizing the interaction between crypto wallet, smart contract, security auditor, and external custody system. The diagram also visualizes the smart contract factory and proxy pattern to anticipate future changes in the security token mechanism. The proposed system architecture can be used as a basic reference to develop an STO mechanism in any blockchain platform. The system architecture proposed in this paper can be modified in the future to fit the exponential growth of blockchain technology. For example, the KYC module in the off-chain system can be replaced by zero-knowledge proof in the privacy-preserving blockchain technology. Blockchain technology allows leaders to independently view the same data. Blockchain will eliminate the need for a central authority so that everyone will have the same ledger, have a consensus mechanism, and can build applications. The problem that is obtained when change management uses its own blockchain technology solution is that the company must also manage security, billing data, and the last problem is to create its own blockchain network which costs quite a lot because it requires its own consultant to build their blockchain network.

## Author Contributions

RR conducts research, data collection, analysis, and article writing. HP, AT, and BS guided, directed, and provided advice on this research. All authors contributed to the article and approved the submitted version.

## Funding

This work is supported by Research and Technology Transfer Office, Bina Nusantara University as a part of Bina Nusantara University.

## Conflict of Interest

The authors declare that the research was conducted in the absence of any commercial or financial relationships that could be construed as a potential conflict of interest.

## Publisher's Note

All claims expressed in this article are solely those of the authors and do not necessarily represent those of their affiliated organizations, or those of the publisher, the editors and the reviewers. Any product that may be evaluated in this article, or claim that may be made by its manufacturer, is not guaranteed or endorsed by the publisher.
